# Molecular and Therapeutic Roles of Non-Coding RNAs in Oral Cancer—A Review

**DOI:** 10.3390/molecules29102402

**Published:** 2024-05-20

**Authors:** Vidhya Rekha Umapathy, Prabhu Manickam Natarajan, Bhuminathan Swamikannu

**Affiliations:** 1 Department of Public Health Dentistry, Dr. M.G.R. Educational and Research Institute, Thai Moogambigai Dental College and Hospital, Chennai 600107, Tamil Nadu, India; 2 Department of Clinical Sciences, Centre of Medical and Bio-Allied Health Sciences and Research Ajman University, Ajman P.O. Box 346, United Arab Emirates; 3 Department of Prosthodontics, Sree Balaji Dental College and Hospital, Pallikaranai, BIHER, Chennai 600100, Tamil Nadu, India; bhumi.sbdch@gmail.com

**Keywords:** oral cancer, transcripts, non-coding RNAs, miRNAs, circular RNAs, long non-coding RNAs, therapeutics

## Abstract

Oral cancer (OC) is among the most common malignancies in the world. Despite advances in therapy, the worst-case scenario for OC remains metastasis, with a 50% survival rate. Therefore, it is critical to comprehend the pathophysiology of the condition and to create diagnostic and treatment plans for OC. The development of high-throughput genome sequencing has revealed that over 90% of the human genome encodes non-coding transcripts, or transcripts that do not code for any proteins. This paper describes the function of these different kinds of non-coding RNAs (ncRNAs) in OC as well as their intriguing therapeutic potential. The onset and development of OC, as well as treatment resistance, are linked to dysregulated ncRNA expression. These ncRNAs’ potentially significant roles in diagnosis and prognosis have been suggested by their differing expression in blood or saliva. We have outlined every promising feature of ncRNAs in the treatment of OC in this study.

## 1. Introduction

Squamous cell carcinoma, which is regularly noted in the oropharynx and oral cavity, is one of the most frequently encountered tumours globally [1]. At an advanced stage, it is very invasive, metastatic, and poses a serious risk to human health [2]. A number of molecular and genetic investigations have provided evidence that links the growth and expansion of squamous cell carcinoma to the accretion of genetic changes at the DNA and RNA levels [3]. Oral squamous cell carcinoma (OSCC) has previously been shown to have genomic alterations (point mutations and copy number variations) and epigenetic changes (methylation and histone modifications), as well as changes in gene expression [3]. Numerous investigations have discovered that the incidence of mutations in prospective cancer genes is higher than anticipated and that distinct combinations of mutations may have an impact on the characteristics of the tumour [4,5,6]. However, worldwide profiling of mutations in OSCC and more knowledge of processes and clinical therapies are hindered by the high cost of Sanger sequencing. The last several years have seen remarkable advancements in sequencing technology that allows for the identification of genetic changes on a genome-wide scale. RNA sequencing (RNA-Seq), a recently discovered deep sequencing method, is widely used in transcriptome profiling. When compared to established techniques like microarray, RNA-Seq provides significantly more accurate transcript expression level measurements and advanced transcript isoform characterisation [7,8]. As a result, it has been effectively used to characterise allele-specific expression patterns and discover genes that express themselves differently [9,10,11,12,13].

More than 90% of the human genome encodes non-coding transcripts, or transcripts that do not code for any protein, according to recent advances in high-throughput genome sequencing [14]. Non-coding RNAs (ncRNAs) were once thought to be useless materials for cells. Advances in thorough molecular examinations have led to a renewed focus on non-coding genes in the development of several disorders, including cancer. tRNA, rRNA, long non-coding RNA (lncRNA), circular RNA (circRNA), microRNA (miRNA), PIWI-interacting RNA (piRNA), and small nucleolar RNA (snoRNA) are the different forms of non-coding RNAs (ncRNAs) that are found in a cell, with different structural and functional properties. It is occasionally the case that the distribution and expression of ncRNAs are tissue- and cell-specific, while at other times, they are universal. It has been discovered that NcRNAs interact with proteins or nucleic acids to change their stabilities, activity, and conformations. It has been shown that distinct tumours have diverse ncRNA expression patterns. MiR-15/16, miR-29, miR-34, miR-200 family, let-7, miR-21, miR-155, miR-17–92 cluster, miR-221/222, miR-195, and miR-26b, for instance, were found to be significantly modulated in a variety of cancers, including leukaemia, prostate, colorectal, pancreatic, liver, lung, breast, glioblastoma, ovarian, renal, and thyroid cancer. These genes are also implicated in the progression, metastasis, and drug resistance of various cancers [15,16]. It has been discovered that treatment resistance in breast cancer is linked to the upregulation of the common target gene Semaphorin 6D (SEMA6D) and the high expression of miR-195 and miR-26b. As a result, this signalling axis is proposed as a predictive marker for the chemotherapy response [16]. Similarly, several cancer types, such as lung, ovarian, prostate, breast, colorectal, gastric, and liver cancers, were seen to have upregulation of the lncRNA HOTAIR and MALAT1, downregulation of the lncRNA Meg3, and a dual function of the lnRNA H19 [15]. Among the circRNAs, it was discovered that glioma, lung cancer, breast cancer, colorectal cancer, gallbladder cancer, gastric cancer, and ovarian cancer all had elevated levels of circPRKCI and circHIPK3 [15]. PiR-651, piR-823, and piR-932 have been shown to be expressed differently in lung, breast, colorectal, oesophageal, and gastric malignancies [15]. Differential expression of non-coding RNAs (ncRNAs) in bodily fluids such as blood and saliva, in addition to primary tissues, indicates their potential utility as diagnostic and therapeutic biomarkers. Our goal in this article is to discuss the traits and roles of RNA transcripts and non-coding RNAs (nc RNAs) associated with oral cancer (OC). In this regard, we list the molecular roles of nc RNAs in cellular processes, present the existing status of ncRNA biomarker discovery, and evaluate the increasing roles of miRNAs, lnc RNAs, circRNAs, and extracellular RNAs in the pathophysiology of OC.

## 2. RNA-Seq-Associated Biomarkers in OC

RNA sequencing (RNA-Seq) is a popular technique for forming a “snapshot” profile of the whole transcriptome. RNA-Seq is a powerful tool for detecting gene transcripts and evaluating genes that exhibit differential expression across a range of disease conditions since it captures the whole transcriptome. To compensate for the shortcomings of malignant transformation prediction, biomarkers have been investigated based on the basic understanding of the molecular aetiology of OSCC. However, though several specific biomarkers have been investigated, none have been proven to be useful in therapeutic settings. The investigation of differential gene expression (DGE) between normal and diseased tissue can provide a comprehensive knowledge of the genetic pathways implicated in the development of cancer. Research with DGE has made it feasible for scientists to analyse the cancer transcriptome in a manner that was not achievable with traditional molecular biology techniques. The paradigm change from using single biomarkers to using gene expression profiles for the diagnosis or prognosis has also been aided by DGE. By defining novel molecular cancer subtypes that improve patient care and targeted therapy, researchers have been able to find patient subgroups with comparable molecular patterns in a variety of tumour forms. One compelling rationale to conduct DGE-based investigations to find gene signatures for early diagnosis, treatment, or prognosis in oral potentially malignant disorders (OPMDs) is the death of prognostic biomarkers in the disease, which would influence focused therapy. When compared with models utilising clinico-pathological risk variables, gene-expression-based prediction models developed by Saintigny et al. show a higher prognostic accuracy [17]. Therefore, more research comparing OPMDs that experience malignant transition to those that do not will shed much-needed light on the molecular processes underlying OPMDs’ malignant transformation. Because whole-transcriptome analysis gives researchers a complete picture of the transcriptional profile at a certain point in time, it represents a significant breakthrough in the study and comprehension of gene expression [17,18,19,20]. Muzamil Khan et al. has found numerous unique DGEs that are linked to the malignant transformation of OPMDs using RNA-Seq technology [21]. Microarray technology was employed in the single prior investigation that evaluated DGE in OPMD in a comparable manner. CYP19A1, HIST1H2AJ, CCDC129, and MUC16 are the four genes found to be identical, while the majority of the statistically significant genes do not match the gene list of 2182 from Saintigny et al.’s research [17].

Cancer-specific non-coding RNAs (ncRNAs) may be useful as biomarkers and therapeutic targets to help customise treatment plans for individuals or patient subgroups. Protein biomarkers and metabolic products are examples of recent medical applications that employ traditional tumour indicators; however, emerging research suggests that non-coding RNAs (ncRNAs) may be better agents for cancer detection and treatment. This is due to the fact that ncRNAs simultaneously target many druggable and non-druggable sites as well as signalling processes. They also have a significantly more stable structure, tissue selectivity, additional tissue-associated activity, and unique RNA properties that can be quickly detected [22]. ncRNAs have been used in a number of clinical studies, particularly as therapeutic and diagnostic biomarkers [23]. By employing antisense oligonucleotides (ASOs) to directly target gene sequences, and by utilising siRNA-associated therapeutic applications, the most advanced and succinct therapeutic approaches for RNA screening have been accomplished thus far [23]. In clinical studies for bladder, pancreatic, and ovarian cancer, the lncRNA H19 promoter sequence was added to the BC-819 plasmid together with the coding sequence of the diphtheria toxin [24]. For thyroid and colorectal cancer diagnostic biomarker investigations, the lncRNAs HOTAIR and CCAT1 (ClinicalTrials.gov) are undergoing clinical trials, and inhibitors of LINC01212 and lncMyoD are being employed as therapeutic markers for melanoma and sarcoma treatment [24]. Each of these research streams suggests that ncRNAs may have therapeutic consequences for cancer treatment and diagnosis. To assess the sensitivity and specificity of miRNA-412 and miR-512 in salivary extracellular vesicles in the malignant development of OC, a clinical investigation has been started. To find diagnostic and prognostic miRNA biomarkers from head and neck cancer tissue, saliva, and blood samples, a new experiment has again been started. Saliva samples from individuals with OC have been used to study the significance of lncRNA MALAT1 and its target miR-124 for both diagnostic and therapeutic purposes. To determine the salivary and plasma miRNAs of patients with head and neck cancer and track changes in these miRNAs during a dietary intervention, a randomised phase II trial has been started. Two investigations have been conducted to evaluate the relationship between blood and plasma miRNA profiles and immunotherapy for head and neck cancer. The companion diagnostic biomarker of OC is low EGFR-AS1 lncRNA expression. A phase II clinical investigation has been initiated to assess the treatment effectiveness of a specific medication of EGFR-associated advanced OC in patients with low EGFR-AS1 OC. The study’s findings may highlight the significance of EGFR-AS1 in OC therapy from the diagnostic and therapeutic standpoints.

## 3. Non-Coding RNA and Cancer

The discovery of ncRNA expanded our knowledge of biological processes. MicroRNAs (miRNAs), long non-coding RNAs (lncRNAs), circular RNAs (circRNAs), and intronic RNAs are all included under the term non-coding RNAs (ncRNAs) [25,26,27]. The functions and molecular mechanisms of ncRNAs are provided in the Supplementary Tables (Tables S1–S3) According to the literature, these RNAs may not be able to encode proteins or peptides. Cancer research has shown that abnormalities in the non-coding genome can result in vital cancer characteristics, in addition to the most well-known protein-coding mutations [28,29]. High-risk HPV infection has recently been identified as a unique risk factor in some patient populations exposed to OC. Studies on cancer have traditionally focused on the expression of messenger RNAs (mRNAs), which are genes that code for proteins. This approach is being scrutinised in light of the finding that just a minor portion of the human transcriptome codes for proteins. According to Ensemble1 (v76) data, only 34% of the human transcriptome codes for proteins, while the remaining 66% is made up of non-coding genes such as pseudogenes, long intergenic non-coding RNAs, antisense RNAs, and miRNAs. Additionally, new research has shown a crucial function for ncRNAs in nearly every stage of the gene expression process, including cellular physiologic activities and the emergence of several human disorders, including cancer [30]. Hence, an opportunity to comprehend the underlying biological processes involved in many malignancies, including OSCC, is presented by knowledge of the roles of various ncRNAs, such as circRNAs, miRNAs, and lncRNAs. This knowledge may eventually result in the creation of brand-new treatments and diagnostic instruments.

miRNAs have been shown to play essential roles in the pathophysiology of several disorders as well as in normal development, differentiation, and growth [31]. Additionally, they are crucial to the development and spread of cancer [31]. miRNA-26a and miRNA-26b’s function in OSCC cells was determined using PCR-based array techniques [32]. Through the control of TMEM184B, the loss of tumour-suppressive miRNA-26a/b in OSCC promotes cancer cell motility and invasion [32]. The researchers concentrated specifically on the category “Regulation of actin cytoskeletal pathway” based on the characteristics of cancer cells after silencing TMEM184B or inducing miR-26a/b. Using GEO expression data, they examined the expression levels of 11 genes related to the “Regulation of actin cytoskeleton pathway.” Among these, si-TMEM184B transfectant cells decreased the expression of three genes (IQGAP3, EGFR, and ITGB4) that were elevated in clinical specimens. The miR26a/b-TMEM184b axis in OSCC cells was shown to be regulated by three genes that were downstream oncogenic genes, according to these results. Cancer cells develop new morphological features and the capacity to cross tissue borders as metastasis progresses. The three members of the Ras GTPase-activating-like protein (IQGAP) family (IQGAP1-3) are involved in cell adhesion, migration, and invasion, among other biological activities. Accordingly, the results showed that miR-26a/b downregulation promoted cancer cell migration and invasion by upregulating TMEM184B expression and activating actin cytoskeleton pathways [32]. Consequently, the tumour-suppressive miR-26a/b-TMEM184B axis may be used as a target for cancer metastasis therapy.

In OSCC, miRNAs were also found in the extracellular vesicles. Here, exosomes generated from OSCC under hypoxic circumstances were shown to include miRNA-21, which markedly increased the expression of vimentin and Snail while markedly lowering levels of E-cadherin in both in vitro and in vivo experiments [33]. Furthermore, in patients with OSCC, circulating exosomal miRNA-21 levels were linked to T stage, lymph node metastases, and HIF-1α/HIF-2α expression [33]. These results imply that tumour cells may be stimulated by a hypoxic microenvironment to produce miRNA-21-rich exosomes, which are then transferred to normoxic cells to encourage prometastatic activities. Because saliva is a commonly accessible biofluid, biomarker techniques using salivary exosomes and/or ex-RNAs are beginning to emerge. In addition, compared to other examined miRNAs, miRNA-27b demonstrated greater sensitivity and specificity in identifying OSCC [34]. Regardless of the size of the tumour, one study discovered that salivary miRNA-31 was considerably raised in all stages of OSCC [35]. Additionally, saliva had greater amounts of miRNA-31 than plasma did, indicating that miRNA-31 is produced locally at the tumour location. Salivary miRNA-31 was remarkably decreased following the removal of OC, indicating that the majority of the salivary miRNA-31 that had been elevated originated from tumour tissues [35]. In total, 509 mRNA core transcripts were found in human-saliva-derived exosomes, according to transcriptome analysis. Exosomes are essential for horizontal gene transfer, as demonstrated by an experiment wherein salivary exosomes were co-cultured in vitro with human oral keratinocytes and changed the recipient cells’ gene expression [36]. The exosome quantity, size, and inter-exosome space were all shown to be increased in oral cancer patients’ saliva, according to a different study [37]. It is interesting to note that exosomes from OC have aberrant shapes and notably higher CD63 surface densities. According to the research, the RNA content of exosomes may provide novel OC biomarkers as well as a useful resource for diagnostics. Exosomes’ specific methods of action in the onset and development of OSCC remain unknown, though.

The primary indicator of cancer is aberrant cell proliferation, which also happens to be the main cause of carcinogenesis. In order to strike a balance between promoting and inhibiting cell growth, extracellular signal molecules and intracellular programmes regulate the specifics of cell cycle development. When cell growth or division spirasl out of control, cells can turn malignant. Years of research have shown that some miRNAs integrate functionally into several important pathways for cell proliferation, and that the dysregulation of these miRNAs is what allows cancer cells to continue proliferative signalling while avoiding growth suppressors.

A family of transcription factors called the E2F proteins plays a crucial role in controlling cell proliferation in a way that is dependent on the cell cycle. Numerous investigations have shown that miRNAs are involved in the control of E2F expression. Since E2F1-deficient mice developed a wide range of malignancies, the E2F member E2F1 was characterised as a tumour suppressor. It promotes target gene transcription during the G1 to S transition [38]. miR-17–92 suppresses E2F1 translation after c-Myc activates it, as demonstrated by O’Donnell et al. [39]. The miR-17–92 cluster may function as a brake on this potential positive feedback loop as c-Myc also directly stimulates E2F1 expression, preventing an abrupt increase in E2F1 protein levels in response to c-Myc activation [40]. It has also been discovered that the miR-17–92 cluster controls the translation of E2F2 and E2F3 [41]. The E2F transcription factors have the ability to stimulate the expression of the miR-17–92 cluster [42]. As a result, under typical circumstances, the feedback loop between the miR-17–92 cluster and E2F offers a way to maintain regular cell cycle progression. But miR-17–92 overexpression, which is prevalent in a number of tumours, shatters the feedback loop that encourages cell division [43].

Different cyclins, cyclin-dependent kinases (Cdks), and their inhibitors are required for cell cycle progression, and miRNAs play a major role in regulating these processes. The first proof that Drosophila germline stem cells with Dicer-1 deletion are inhibited in the G1/S transition was given by Hatfield et al. [44]. This suggests that miRNAs are necessary for germline stem cells to cross the typical G1/S checkpoint. Additionally, Dacapo, a member of the p21/p27 family of Cdk inhibitors, was expressed more often in germline stem cells lacking Dicer, suggesting that miRNAs negatively control this protein to encourage cell cycle advancement. Indeed, it has been found that miR-221/222 specifically targets the Cdk inhibitor p27Kip1 in glioblastoma cells [45]. This finding was r verified in original tumour samples and other cancer cell lines [46,47,48]. In cancer cells, ectopic expression of miR-221/222 sped up cell division, while its suppression resulted in G1 cell cycle halt. Furthermore, it has been discovered that the expression of miR-221/222 is elevated in a range of human tumours, indicating that the control of p27Kip1 by miR-221/222 is a legitimate oncogenic pathway. Like p27Kip1, miRNAs like miR-663, the miR-302 family, and miR-24 also influence p21CIP1 and p16INK4a [49,50]. miR-663 was shown to be elevated in nasopharyngeal cancer, and it functions as an oncogene by directly targeting p21CIP1 to promote the cellular G1/S transition both in vitro and in vivo. Thus, the molecular mechanism of nasopharyngeal cancer cell proliferation was elucidated via the miR-663/p21CIP1 axis [51].

## 4. Circular RNAs and Cancer

**Figure 1:**
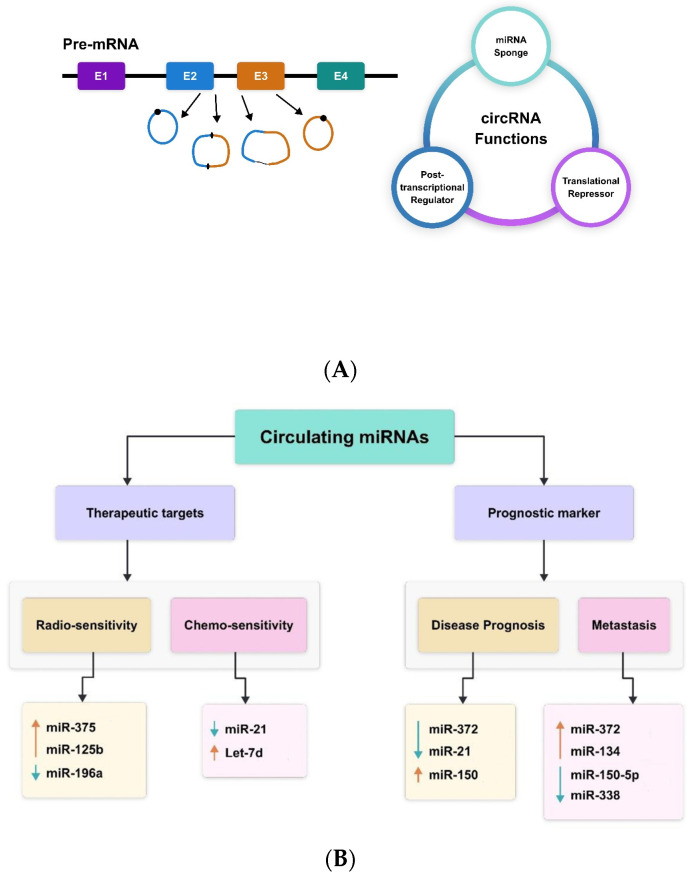
CircRNAs—(**A**) biogenesis and function; (**B**) therapeutic and prognostic biomarkers miR-375 [59], miR-125b [60], miR-196a [61], miR-21 [62], Let-7d [63], miR-372 [53], miR-21 [64], miR-150 [57], miR-372 [53], miR-134 [54], miR-150-5p [57], miR-338 [58,65,66].

A subclass of ncRNAs known as circular RNAs (circRNAs) has very lately come to light as a novel regulator of gene expression [52]. In mammalian cells, they appear in a tissue-specific way and form a covalently closed continuous loop without 5′ caps and 3′ tails. Circular RNAs’ biogenesis and developing function have been well-discussed elsewhere [53,54,55]. Figure 1 presents a summary of the biosynthesis and function of circRNAs. Briefly, RNA polymerase II backsplices genes to create circRNAs from precursor mRNA, and the circRNAs are frequently expressed at low levels [53]. The cis and trans regulatory components that govern splicing are necessary for the regulation of circRNA biogenesis. The majority of circRNAs are made up of multiple exons, often two or three. Furthermore, by using alternative splicing, a single gene can create many circRNAs, either including or excluding the internal intron [53,54,55]. According to recent research, some circRNAs influence gene expression at several levels and have critical physiological roles [56,57]. CircRNAs also have the ability to absorb miRNAs. For example, the vertebrate cerebellar degeneration-related 1 (CDR1) antisense transcript yields a circular RNA sponge for miRNA-7 (ciRS-7), which functions as an RNA sponge for miRNA-7 [58].

Circular intronic RNAs (ciRNAs), formed from lariat introns, are the nuclear circular RNAs found in cells. ciRNAs that are widely expressed, such ci-ankrd52 and ci-sirt7, are found in the nucleus and engage in interactions with the elongating Pol II complex [53]. It has been demonstrated that depleting these ciRNAs reduces the transcription levels of the genes sirtuin 7 and ankyrin repeat domain 52. Although the exact method of action of ciRNAs in transcription control remains unclear, they contribute to Pol II transcription [65]. circRNAs were thought to be the consequence of splicing mistakes with no known function, despite the fact that they were originally identified almost 20 years ago through the identification of spliced transcripts of a putative tumour suppressor [66]. Many circRNAs have been found since sequencing and other genomic techniques were introduced [64]. CircRNAs have several benefits for comprehending and keeping track of regulatory networks. First, because circRNAs are resistant to RNase action, they are more persistent than mRNAs in vivo. Second, circRNAs are more common than linear mRNA, sometimes by more than ten times. Third, the tissue specificity of circRNAs makes them perfect for research on cancer biology and the identification of biomarkers.

## 5. Circular RNAs in OSCC

According to recent research, circRNAs and many cancer types may be tightly related [53]. However, there is not much research on circRNAs in OSCC because this topic is very new. Many circRNAs were found to be differentially expressed between OSCC tissue and paired non-cancerous matched tissue in a recent thorough investigation of circRNAs in human OSCC employing circRNA and mRNA microarrays [53]. An important regulator of OSCC advancement is circRNA_100290. Functional investigations revealed that circRNA_100290 knockdown reduced CDK6 expression, caused G1/S arrest, and slowed the growth of OSCC cell lines. Direct interaction between circRNA_100290 and members of the miRNA-29 family was discovered using luciferase reporter assays [39]. EGFP/RFP reporter assays were utilised to determine the usefulness of taking CDK6 as a direct target of miRNA-29b. These results suggest that circRNA_100290 has a competitive endogenous role in which it regulates CDK6 expression by sponging the miRNA-29b family.

## 6. Long Non-Coding RNA in Cancer

**Figure 2:**
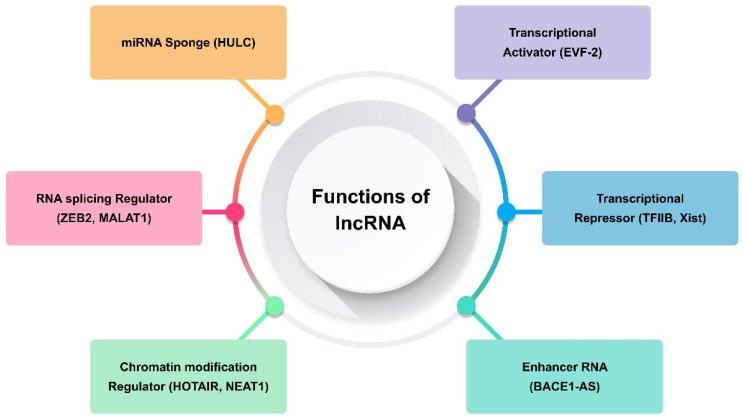
Functions of long non-coding RNA (lncRNA).

Transcripts longer than 200 nt and lacking the ability to code for proteins are referred to as long non-coding RNAs (lncRNAs) [31]. They are only expressed in particular cancer types or differentiated tissues [67]. The transcription of lncRNAs is carried out by RNA polymerase II, and their expression is often tissue-specific. They are fundamental to many biological processes, including development, differentiation, and stem cell biology. It has been demonstrated that human diseases, including many forms of cancer, are intimately linked to lncRNA deregulation [31,67]. Consequently, the present focus of cancer research is on lncRNAs, and a new field of study comprising functional annotation of lncRNAs is developing. Generally speaking, lncRNAs use several methods to carry out their biological activities. Examples of such lncRNAs include those that modify protein complex stability, operate as a sponge for miRNA repression, and alter chromatin remodelling and methylation (Figure 2) [29,68,69]. Certain lncRNAs, such TARID, AS1DHRS4, and Kcnq1ot1, have been shown to either directly recruit DNA methyltransferases to alter chromatin conformation or, in the case of SChLAP1, to adjust nucleosome placement through the SWI/SNF complex [60]. In one study, chromosome inactivation was caused by Polycomb repressive complex-2 (PRC2), which induced inhibitory H3K427me3 histone marks [70]. On the other hand, other lncRNAs, such CCAT1 and HOTTIP, activate chromatin, which modifies chromosomal looping and impacts gene promoters [71,72]. Maintaining X chromosomal inactivation is a function of the lncRNA Firre [73]. One of the established pathways that alters chromatin conformation after X chromosome inactivation is the binding of the X-linked lncRNA Firre with the chromatin remodellers CTCF and cohesin. As a result, H3K27me3 methylation is preserved and the inactive chromosome X is located close to the nucleolus [73]. By attaching to miRNAs, which can sequester these biomolecules and lessen their inhibitory capability on their targets, other lncRNAs bind to their targets and carry out their inhibitory actions [67].

## 7. Long Non-Coding RNA in OSCC

**Figure 3:**
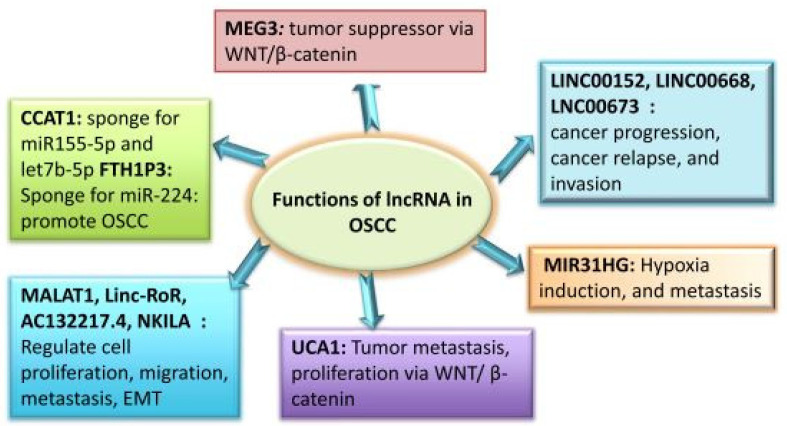
Roles of lncRNAs in OSCC.

Although the functional significance of lncRNAs in OSCC has been thoroughly studied in the past 10 years, it is still unknown how miRNA functions in these tumours. Many lncRNAs, especially those related to the head and neck, are dysregulated in malignancies [33]. It has been determined that OC tumours and normal human tissue have different lncRNA expression patterns. lncRNAs linked to cancer participate in several cellular processes, including proliferation, differentiation, tumour invasion, and metastasis. Some of these lncRNAs function as oncogenes, whereas the remainder function as tumour suppressors. Certain lncRNAs linked with OSCC have been found to have well-defined roles in the development and progression of cancer. These findings imply that OSCC treatment may benefit from the use of these lncRNAs as possible therapeutic targets, as well as new biomarkers and monitoring tools. In the OSCC pathogenesis, lncRNAs play a variety of roles, which are summarised in Figure 3.

## 8. microRNAs (miRNAs)

One family of small, non-coding RNAs with a length of 18–25 nucleotides is known as microRNAs (miRNAs). By attaching to complementary regions in messenger RNA (mRNA), miRNAs—conserved sequences in the genome—control post-transcriptional changes in gene expression [74]. miRNAs are RNA molecules that bind to the 3′ untranslated region (UTR), 5′UTR, or coding regions of target mRNA. This binding can result in translation inhibition or mRNA destruction, and was first seen in Caenorhabditis elegans in 1993 [59]. About 30% of human mRNA transcripts are regulated by miRNAs, which inhibit cell growth, death, and metabolism [63]. Due to their short length, miRNAs are important in both health and diseases since they can target hundreds of mRNAs in the same or a distinct functional pathway. Deregulation of miRNAs, which act as oncogenes or tumour suppressor genes, has been linked to a range of diseases, including schizophrenia, diabetes mellitus, cardiovascular disease, and malignancies including OC [75]. Leukoplakia, erythroplakia, melanoplakia, and submucous fibrosis are examples of oral premalignant lesions (OPMLs) that eventually advance before becoming oral malignancies. Numerous miRNAs are linked to OPMLs and may increase the likelihood that the oral lesions will develop into malignant phenotypes. Therefore, knowing the molecular pathophysiology of the disease may help explain emerging strategies for improving the patient’s prognosis and outcome. The miRNA network may reveal new essential molecules with non-invasive potential as prognostic and diagnostic indicators as well as therapeutic markers for therapy.

## 9. miRNA Deregulation

Epigenetic regulation: Global DNA hypomethylation, aberrant DNA hypermethylation in the promoter region of tumour suppressor genes, and post-translational histone modifications are examples of epigenetic changes that are known to occur in cancer. Like genes that code proteins, a number of miRNA genes that serve as tumour suppressors are regulated epigenetically by hypermethylation, which silences the genes. OC patients’ tissue and saliva exhibited 64.6% promoter methylation of miR-137, which was linked to overall survival [61,62]. Patients with OC also had hypermethylation of miR-124 (40%), miR-193a (72.7%), miR-200c-141 (36%), miR-218 (73.33%), miR-585 (13.3%), and miR-596 (61.25%) [62,76].

Gene deletions: Gene site changes and deletions may be the cause of aberrant miRNA expression in cancerous cells. On chromosome 11 (11q24.1), miR-100 and miR-125b are found in the distal band 11q24 area; oral malignancies frequently exhibit loss of heterozygosity (LOH) in this region [77]. Patients with OSCC have been shown to have lost the RB1 gene and the miR-15a/16-1 cluster on chromosome 13q14. OC is, therefore, linked to the loss of the miR-15a/16-1 cluster and the miR-100 and miR-125b genes.

Modifications in the process of miRNA biogenesis: The production of miRNA is a tightly controlled process, and modifications to the biogenesis pathway impair the activities of miRNA. Abnormal expression of miRNAs can result from mutations or aberrant expression in any of the part of the machinery involved in miRNA production. In OC, downregulation of the tumour suppressor miRNA let-7b and enhanced cell proliferation have been linked to DICER1 overexpression [78]. The expression patterns and clinicopathological consequences of the dysregulation of numerous proteins linked to the machinery of miRNA synthesis, such as Drosha, Exportin 5, DICER, and Argonaute proteins, have not yet been determined in OC, despite the fact that these proteins are dysregulated in a number of cancers.

## 10. miRNAs Deregulated in OC

In OC, there has been an association found between the upregulation of miR 10a, miR-21, miR-24, and miR-196a, the downregulation of miR-26a, miR-30a, and miR-375, and an increase in growth characteristics, including cell growth, proliferation, and cell cycle progression [32,79,80,81,82]. Ancillary independent colony formation is linked to upregulation of miR-21 and miR-211 and downregulation of miR-375; augmented vascular invasion is linked to upregulation of miR-21, miR-155, miR-211, and miR-504. In most cases (79%) of oral malignancies, Lin and colleagues found that miR-24 expression was two times greater [83]. In OC, miR-24 inhibits DND1 expression, which influences CDKN1B [57]. OC is associated with overexpression of miR-155 (2.5-fold), which has a testing accuracy of >90% [84]. Through the epithelial–mesenchymal transition (EMT), miR-155 aids in the invasion of cancer by reducing SOCS1 and increasing STAT3 expression.

## 11. Clinical Relevance of Deregulated miRNAs

Accurate subclassification of oral malignancies is crucial since the physiology and genetic makeup of the subsites in the mouth cavity vary greatly. miRNAs play a crucial role in the establishment and maintenance of gene expression patterns unique to certain tissues. miRNA pathways can affect tissue-specific behaviour and production and are crucial for a variety of tissue-specific cell types. Differential miRNA profiles in OC can support tumours to be categorised according to the tissues from which they originated [85]. The initial lesion and local metastatic disease may be compared using the miRNA expression profile that was preserved between the primary cancer subsite and nodal metastasis [86]. Manikandan et al. found that tongue malignancies expressed more let-7f than gingivo-buccal tumours [87]. A therapeutic strategy for OC can be identified by identifying discrete prognostic groupings based on the identification of subsite-specific miRNAs and their correlation with physiologic function and the OC process.

## 12. miRNAs as Prognostic Biomarkers in OC

Certain miRNAs’ expression patterns have been positively correlated with patient survival, lymph node metastases, and clinical stage; this suggests that certain miRNAs may function as prognostic indicators in OSCC. It has been observed that miR-146a increases metastasis by downregulating TRAF6, NUMB, and IRAK1 expression [88]. However, this is at odds with another work, which showed by focusing on the mRNA for SOX2 that overexpressing miR-146a inhibited invasion, tumorigenicity, and metastasis in OSCC cell lines [89]. Given that miR-46a is said to exhibit biphasic expression, analysis of the deregulation of downstream mRNAs is necessary to make judgements on its prognostic and diagnostic use in OSCC. In OSCC, miR-338 is a tumour-suppressor miRNA as well. The cancer prognosis and metastatic risk may be associated with both the miRNAs and their target mRNAs that are abundant in a certain tissue type, as a single miRNA targets numerous mRNAs.

## 13. Circulating miRNAs’ Potential Therapeutic Use in OC

Recently, there has been a lot of interest in the potential innovative therapeutic method of manipulating miRNA expression and function through the local and systemic administration of miRNA inhibitors or miRNA mimics [90]. The benefit of miRNA-based cancer treatment is its capacity to simultaneously target many effectors of pathways related to cell survival, proliferation, and differentiation. The main difficulties, meanwhile, lie in delivering these polyanionic oligonucleotides into living things. Naked miRNAs are vulnerable to RNase degradation and are unable to cross hydrophobic cell membranes. Both chemically altered miRNA-targeting antisense oligonucleotides and delivery methods based on nanoparticles have demonstrated significant potential in enhancing the stability and bioavailability of miRNAs. Nonetheless, effective targeting to certain bodily parts continues to be difficult [91].

One of the biggest problems in treating patients with OSCC is resistance to chemotherapy and radiation. Recent research has connected changed miRNA expression with OSCC resistance to chemotherapy and radiation. Patients with OSCC have plasma levels of the tumour suppressor miRNA miR-375 that are reportedly lower than normal [92]. It has been demonstrated that by inhibiting IGF1R, miR-375 reduces proliferation and increases the radiosensitivity of OSCC. Since low miR-375 expression may be linked to radiation resistance, overexpressing miR-375 via miRNA mimics may improve OSCC patients’ sensitivity and responsiveness to radiation. Patients with OSCC had reduced blood levels of miRNA Let-7d, another tumour suppressor. Moreover, patients with OSCC exhibited increased chemoresistance when this miRNA’s expression was reduced [93]. Therefore, it is anticipated that patients would become more chemosensitive and responsive to chemotherapy as a result of the targeted distribution of Let-7d mimics. Nonetheless, even though miRNAs have shown promise as therapeutic agents for cancer, more thorough investigations and studies are needed to create an efficient in vivo delivery method that will allow miRNAs to be targeted and absorbed to their maximum potential.

## 14. Functions and Mechanisms of circRNA/lncRNA Interaction with miRNA

CircRNA and lncRNA interact with miRNAs through a variety of methods to perform their functions. The mechanisms behind the relationship between lncRNA and miRNA are as follows: lncRNA sponges up miRNA, lncRNA competes with miRNA for mRNA, miRNA recognises RISC and regulates lncRNA, and lncRNAs are precursors of miRNAs. Sponge adsorption, storage and transport, and expression interference comprise the interaction process between circRNA and miRNA. One of the most efficient ways to control miRNA is through the sponge adsorption mode, which is shared by circRNA and lncRNA. Nowadays, miRNA research frequently concentrate on miRNA sponges that influence miRNA expression. Like a sponge absorbing water, miRNA sponges may absorb a large number of miRNAs. Four to ten miRNA binding sites make up a typical miRNA sponge, and each one has mismatches at intermediate places to prevent them from being impacted by argonaute 2 (Ago2)’s endoribonuclease activity [94]. Complementary sponges bear a resemblance to short interfering RNAs (siRNAs), which possess comparable suppression properties but are prone to destruction. miRNA sponges are able to efficiently block miRNA clusters with a common seed sequence and can target a single miRNA or several distinct miRNAs [95]. miRNA sponges address the issue of type III promoters not being expressed in particular cells and are not biased in their promoter selection. [96]. Furthermore, a single long-stranded sponge may express numerous miRNA binding sequences concurrently, making it more stable in terms of inhibition duration and impact and allowing it to knock down many miRNAs at once [97]. Certain antisense sequences of miRNAs, such hsa-miR-548bb-5p, have four consecutive Ts. Type III promoters like U6 and H1 experience inadequate transcription as a result of these four consecutive Ts, which results in the creation of their repressor codon sequences. On the other hand, miRNA sponges can address the issue of miRNAs with these repressive codon sequences as they lack a bias for promoter selection. Furthermore, the mismatched form of miRNA sponges is produced to prevent cleavage of its adsorption sequences. Furthermore, polyadenosine (polyA) tails are present in miRNA sponges produced by type II promoters, which have a longer half-life and a more consistent inhibitory impact than inhibitors [98].

## 15. Conclusions

This review has outlined the significant contribution of quantitative and qualitative changes to several ncRNAs (circRNA, lncRNA, and miRNA) in the onset of OC. The ncRNAs control metastasis, drug resistance, cell survival, proliferation, and angiogenesis simultaneously by targeting various signalling molecules. MiRNA expression profiles have been shown through genome-wide profiling to be associated with the tumour type, tumour grade, and clinical outcomes. Therefore, miRNAs may be useful as prognostic or diagnostic biomarkers, therapeutic targets, or instruments. To evaluate miRNA candidates using deep sequencing and verify them as predictive and diagnostic biomarkers in a sizable patient sample cohort, further work is still required. The following problems need to be resolved in order to create miRNA treatment strategies: the creation of an effective and targeted miRNA delivery system; the validation of the targets; and the precise prediction of any undesirable off-target effects. Without a doubt, more research into the new miRNAs, their biological activities, and the genes that they target will expand our understanding of the roles miRNAs play in tumorigenesis and support the development of miRNA-related cancer prognosis, diagnostics, and therapy. Research has shown a correlation between the high rate of morbidity and death in OC and the difficulty of making an accurate diagnosis and providing the right care. Prompt diagnosis can be essential for preventing OPMD from spreading and invading other areas of the body, as well as for extending the lives of those affected. In light of this, the regulatory role of various ncRNA profiles is receiving a lot of attention since it shows great potential for use in recognising OC lesions.

## Data Availability

The data presented in this study are available in this article.

## Supplementary Materials

The following supporting information can be downloaded at: https://www.mdpi.com/article/10.3390/molecules29102402/s1, Table S1. Functions and molecular mechanism of miRNAs regulating oral cancer. Table S2. Functions and molecular mechanism of lncRNAs regulating oral cancer. Table S3. Functions and molecular mechanism of circRNAs regulating oral cancer. References in SM file [99,100,101,102,103,104,105,106,107,108,109,110,111,112,113,114,115,116,117,118,119,120,121,122,123,124,125,126,127,128,129,130,131,132,133,134,135,136,137,138,139,140,141,142,143,144,145,146,147,148,149,150,151,152,153,154,155,156,157,158,159,160,161,162,163,164,165,166,167,168,169,170,171,172,173,174,175,176].
